# Integration of radiation oncology teaching in medical studies by German medical faculties due to the new licensing regulations: An overview and recommendations of the consortium academic radiation oncology of the German Society for Radiation Oncology (DEGRO)

**DOI:** 10.1007/s00066-021-01861-7

**Published:** 2021-11-16

**Authors:** H. Dapper, C. Belka, F. Bock, V. Budach, W. Budach, H. Christiansen, J. Debus, L. Distel, J. Dunst, F. Eckert, H. Eich, W. Eicheler, R. Engenhart-Cabillic, R. Fietkau, D. F. Fleischmann, B. Frerker, F. A. Giordano, A. L. Grosu, K. Herfarth, G. Hildebrandt, D. Kaul, O. Kölbl, M. Krause, D. Krug, D. Martin, C. Matuschek, D. Medenwald, N. H. Nicolay, M. Niewald, M. Oertel, C. Petersen, F. Pohl, A. Raabe, C. Rödel, C. Rübe, C. Schmalz, L. C. Schmeel, D. Steinmann, G. Stüben, R. Thamm, D. Vordermark, H. Vorwerk, T. Wiegel, D. Zips, S. E. Combs

**Affiliations:** 1 Department of Radiation Oncology, Technical University of Munich, Munich, Germany; 2 Department of Radiation Oncology, LMU University Hospital, Munich, Germany; 3 Department of Radiation Oncology, Rostock University Medical Center, Rostock, Germany; 4 Corporate Member of Freie Universität Berlin, Humboldt-Universität zu Berlin, and Berlin Institute of Health, Department of Radiation Oncology, Charité—Universitätsmedizin Berlin, Berlin, Germany; 5 Department of Radiation Oncology, Medical Faculty, Heinrich Heine University, Duesseldorf, Germany; 6 Department of Radiation Oncology, Hannover Medical School (MHH), Hannover, Germany; 7 Department of Radiation Oncology, Heidelberg University Hospital, Heidelberg, Germany; 8 Department of Radiation Oncology, University Hospital Erlangen, Erlangen, Germany; 9 Department of Radiation Oncology, University Hospital Schleswig-Holstein, Kiel, Germany; 10 Department of Radiation Oncology, University of Tübingen, Tübingen, Germany; 11 Department of Radiation Oncology, University of Münster, Münster, Germany; 12 OncoRay—National Center for Radiation Research in Oncology, Faculty of Medicine and University Hospital Carl Gustav Carus, Technische Universität Dresden, Helmholtz-Zentrum Dresden—Rossendorf, Dresden, Germany; 13 Department of Radiotherapy and Radiation Oncology, University of Marburg, Marburg, Germany; 14 Department of Radiation Oncology, University Hospital Bonn, University of Bonn, Bonn, Germany; 15 Department of Radiation Oncology, University Medical Center Freiburg, Freiburg, Germany; 16 Corporate Member of Freie Universität Berlin, Humboldt-Universität zu Berlin, and Berlin Institute of Health, Department of Radiation Oncology, Charité—Universitätsmedizin Berlin, Berlin, Germany; 17 Department of Radiotherapy, University of Regensburg, Regensburg, Germany; 18 Department of Radiation Oncology, Faculty of Medicine and University Hospital Carl Gustav Carus, Technische Universität Dresden, Dresden, Germany; 19 Department of Radiotherapy and Oncology, University Hospital, Goethe University, Frankfurt, Germany; 20 Deptartment of Radiation Oncology, Martin Luther University Halle-Wittenberg, Halle (Saale), Germany; 21 Department of Radiotherapy and Radiooncology, Saarland University Medical Center, Homburg, Germany; 22 Department of Radiotherapy and Radio-Oncology, University Medical Center Hamburg Eppendorf, Hamburg, Germany; 23 Department of Radiation Oncology, University of Augsburg, Augsburg, Germany; 24 Department of Radiation Oncology and Radiotherapy, University Hospital Ulm, Ulm, Germany; 25 Department of Radiation Oncology, Technical University of Munich, Munich, Germany; 26 German Cancer Consortium (DKTK) Partner Site (DKTK), Munich, Germany; 27 German Cancer Research Center (DKFZ), German Cancer Consortium (DKTK), Heidelberg, Germany; 28 Heidelberg Ion-Beam Therapy Center, Heidelberg, Germany; 29 German Cancer Consortium (DKTK) Partner Site (DKTK), Tübingen, Germany; 30 German Cancer Consortium (DKTK) Partner Site (DKTK), Freiburg, Germany; 31 Partner Site Berlin, German Cancer Research Center (DKFZ), German Cancer Consortium (DKTK), Heidelberg, Germany; 32 OncoRay—National Center for Radiation Research in Oncology, Faculty of Medicine and University Hospital Carl Gustav Carus, Technische Universität Dresden and Helmholtz-Zentrum Dresden—Rossendorf, Dresden, Germany; 33 Partner Site Dresden, German Cancer Research Center (DKFZ), National Center for Tumor Diseases (NCT), Heidelberg, Germany; 34 Helmholtz-Zentrum Dresden—Rossendorf, Dresden, Germany; 35 Heidelberg and German Cancer Consortium (DKTK), German Cancer Research Center (DKFZ), Dresden, Germany; 36 German Cancer Consortium (DKTK) Partner Site (DKTK), Frankfurt, Germany; 37 Institute of Radiation Medicine, Department of Radiation Sciences, Helmholtz Zentrum München, Munich, Germany; 38 German Cancer Consortium (DKTK) Partner Site (DKTK), Munich, Germany

**Keywords:** Radiation oncology teaching, Medical studies, New licensing regulations

## Abstract

The new Medical Licensing Regulations 2025 (*Ärztliche Approbationsordnung, ÄApprO*) will soon be passed by the Federal Council (Bundesrat) and will be implemented step by step by the individual faculties in the coming months. The further development of medical studies essentially involves an orientation from fact-based to competence-based learning and focuses on practical, longitudinal and interdisciplinary training. Radiation oncology and radiation therapy are important components of therapeutic oncology and are of great importance for public health, both clinically and epidemiologically, and therefore should be given appropriate attention in medical education. This report is based on a recent survey on the current state of radiation therapy teaching at university hospitals in Germany as well as the contents of the National Competence Based Learning Objectives Catalogue for Medicine 2.0 (*Nationaler Kompetenzbasierter Lernzielkatalog Medizin 2.0, NKLM*) and the closely related Subject Catalogue (*Gegenstandskatalog, GK*) of the Institute for Medical and Pharmaceutical Examination Questions (*Institut für Medizinische und Pharmazeutische Prüfungsfragen, IMPP*). The current recommendations of the German Society for Radiation Oncology (*Deutsche Gesellschaft für Radioonkologie, DEGRO*) regarding topics, scope and rationale for the establishment of radiation oncology teaching at the respective faculties are also included.

## Background and design of the new medical licensing regulations (ÄApprO)

With the Masterplan Medical Education 2020 *(Masterplan Medizinstudium* 2020) from 31 March 2017, the Health and Science Ministers of the federal and state governments adopted a resolution comprising 37 measures to restructure and modernize medical studies in Germany [1]. The experience gained in the further development of medical studies from the model study programs at individual universities was incorporated with an emphasis on practice-oriented, longitudinal and interdisciplinary training [2]. The main focus is on changing the orientation of the study program from fact-based to competence-based learning. Medical students and expert groups have been calling for a corresponding redesign of medical studies for some time [3, 4].

The content of the Masterplan Medical Education 2020 is defined by the National Competence-Based Learning Objectives Catalogue for Medicine 2.0 (NKLM), which has been developed as a cooperative project of the Medical Faculty Association of the Federal Republic of Germany (*Medizinischer Fakultätentag der Bundesrepublik Deutschland, MFT*) and the Society for Medical Education (*Gesellschaft für Medizinische Ausbildung, GMA*) as an ongoing process since 2015; and the closely related Subject Catalogue (GK) of the Institute for Medical and Pharmaceutical Examination Questions (IMPP) [5, 6].

**Fig. 1:**
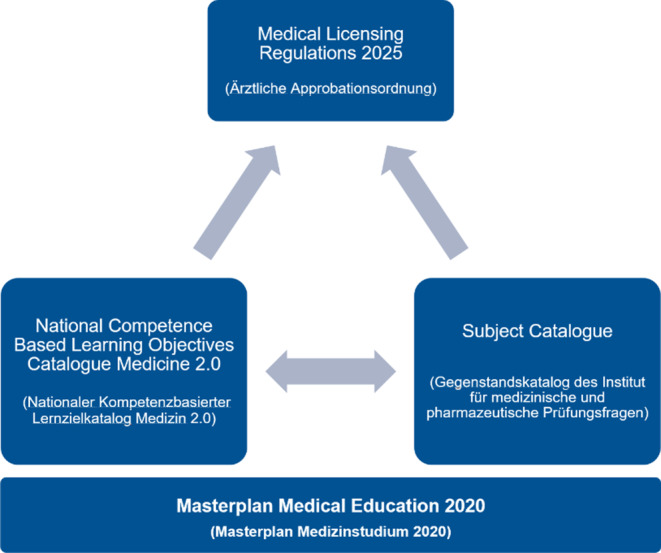
Development process of the new Medical Licensing Regulations (Ärztliche Approbationsordnung), which will finally be enacted by the Federal Ministry of Health in 2025. The 2nd draft of the Medical Licensing Regulations was created at the end of 2020 against the background of the Masterplan Medical education 2020 (*Masterplan Medizinstudium *2020). This draft is essentially based on the National Competence-Based Learning Objectives Catalogue for Medicine 2.0 (*Nationaler Kompetenzbasierter Lernzielkatalog Medizin* 2.0) drafted by the Medical Faculty Association of the Federal Republic of Germany (*Medizinischer Fakultätentag der Bundesrepublik Deutschland*) and the Society for Medical Education (*Gesellschaft für Medizinische Ausbildung*) in cooperation with the Subject Catalogue (Gegenstandskatalog) of the Institute for Medical and Pharmaceutical Examination Questions (*Institut für Medizinische und Pharmazeutische Prüfungsfragen*)

Due to this further development, the reforms will result in the new Medical Licensing Regulations (ÄApprO), which will finally become effective in 2025 (Fig. 1). The current draft of the ÄApprO is expected to be passed by the Bundesrat in the next few months [7]. Its contents are, depending on the faculty, already being implemented at present or will be implemented step-by-step at the various sites in the coming months.

In addition to a competency orientation, i.e., an increasing differentiation between knowledge and practical skills, a longitudinal structure of the curriculum (Z-Curriculum) is the guiding principle. Consequently, the strict separation between preclinical and clinical as well as between individual medical subjects is eliminated. The main cornerstones, orientations and innovations are:

- Structuring of the study program based on the NKLM into a core area (approx. 80%) and a specialization area (approx. 20%), which is chosen by the students, which differs among the universities
- Increase of patient-centered teaching (e.g., clinical placements, actual patient cases, some of which may be simulated) and digital teaching formats
- Division into basic sciences, clinical subjects and higher-level competencies
- Outcome-oriented learning and longitudinal organization of studies in modules with interdisciplinary, competence-based final examinations for each module (the university defines the modules including the subjects and examinations included)
- distribution of the total workload into fixed teaching hours (14,400 teaching units; 1 teaching unit = 45 min)
- Strengthening of general medicine and public health services
- Division of the final practical year into quarters (incl. compulsory quarter in general medicine) and requiring a scientific paper between the 1st and 2nd state examinations

The draft by the Federal Ministry of Health was assessed as fundamentally positive by Hartmannbund, Marburger Bund and the Federal Representation of Medical Students in Germany (*Bundesvertretung der Medizinstudierenden in Deutschland, bvmd*) [8, 9].

### Significance of the new medical licensing regulations for radiation therapy and radiation oncology

In the current draft of the ÄApprO, radiation therapy is integrated into the cross-sectional subject “imaging procedures, radiation therapy, radiation protection” (*Bildgebende Verfahren*) [1] and is rarely explicitly mentioned as an individual subject in the NKLM as well as in the GK (see GK VII.4.16) [5, 6]. Due to the still pending specific implementation of the longitudinal curriculum, which is the responsibility of the respective medical faculties, the scope, positioning and implementation of radiation therapy teaching is still open. In view of the relatively strictly defined number of total teaching units, the longitudinal curriculum structure, and the extensive detachment of teaching subjects, the debates among representatives from individual subjects regarding the share of teaching units at the faculties will probably increase.

However, due to the topic-related teaching and the focus on competence creation and the associated elimination of the clear assignment of topics to specific subjects (e.g. rectal cancer to surgery), there are also extensive opportunities for a strong representation of interdisciplinary radiation oncology to be included in the new curricula. Promising integration possibilities of radiation therapy teaching arise in the topics of the most frequent tumor entities, which are mostly very strongly weighted in the NKLM and the GK, medical interview management, basic sciences and, of course, in the area of the cross-sectional subject (QS) imaging. For example, a longitudinal module “interdisciplinary oncology” is conceivable in the future, in which students acquire basic competencies for individual tumor entities in the form of guided self-study, instructional videos, seminars, lectures, and case discussion rounds, and then work out an interdisciplinary treatment plan, including radiation oncology, in a case-based seminar according to the “flipped classroom” model. Although radiation therapy and radiation oncology do not occupy an overriding role in the context of overall medical education, they are of great importance clinically as well as in terms of health economics and epidemiology. Actually, almost every specialty has intersections with radiation therapy, and about half of the oncology patients receive radiation therapy treatment during the course of their disease [10]. The present concept paper contains the current DEGRO recommendations for the establishment of radiation therapy teaching at the respective faculties and essentially answers the questions:

1. Which radiation therapy topics should be taught as a minimum and to what extent?
2. At which point and in which teaching format can these topics be anchored in the new curriculum?
3. What are the arguments for establishing radiation therapy teaching in the respective faculties?

## Which radiation therapy topics should be taught and to what extent?

### Survey of radiation oncology faculty teaching

In April 2021, the teaching staff and heads of 21 university hospitals for radiation therapy and radiation oncology participated in a survey of the Academic Working Group Radiation Oncology (*AG Akademische Radioonkologie*, AKRO of DEGRO) on the current state of radiation oncology teaching at the respective university hospitals. Current teaching formats, both mandatory and elective, were queried and quantified. Furthermore, the current status of virtual instruction and possible requests for optimization of teaching were queried. The results of the survey are summarized in Table 1.

**Table 1:** Results of survey teaching radiation oncology at the faculties, *n* = 21

**Current compulsory scope of teaching in the curriculum**			
*Primary semester in which teaching takes place (median)*	6 (median)		
*Current subject integration of radiation oncology*	QS imaging	Separate subject	Other
	**13 **(62%)	**3 **(14%)	**5 **(21%)
*Current compulsory courses (UE/student/study) *(median/range)	Lecture	Seminar	Bedside teaching/patient contact
	**10 **(0–21)^a^	**8 **(0–20)^a^	**1 **(0–12)
*Current sufficient amount of teaching*	Yes	No	–
	**5** (24%)	**16** (76%)	–
**Current optional course offerings in the curriculum**			
*Optional courses*	Elective subject	PJ-tertial	Other
	**17 **(81%)	**15** (71%)	**9 **(43%)
**Digitization of radiation oncology teaching**			
*Current implementation of virtual teaching*	Yes	No	–
	**20** (95%)	**1** (5%)	–
*Courses in which virtual teaching is currently carried out*	Seminar	Lecture	Other
	**14** (67%)	**18 **(86%)	**5** (24%)
*Virtual teaching in the future (after COVID 19 pandemic)*	Yes, as currently	Yes + expansion	No
	**10 **(48%)	**8 **(38%)	**3 **(14%)
**Optimization possibilities of radiation oncology teaching**			
*Recommendations regarding the optimization of radiation oncology teaching*	More UE		**16** (76%)
	Interdisciplinary, longitudinal teaching (free text)		**14** (67%)
	Other (free text: PJ-tertial, elective, more face-to-face teaching, etc.)		**7** (33%)

*QS* cross-sectional subject, *UE* teaching unit (45 min), *PJ* practical year

^a^Participation in interdisciplinary lectures/seminar series was counted as 0.25 UE

At most sites, teaching still takes place in the classical curriculum with strict separation of clinical and preclinical subjects, and mainly within the QS imaging (62%) [11, 12]. Most of the teaching takes place in the early clinical semesters (median 6th semester), due to the routine assignment to the introductory QS Imaging. This is not considered useful by most of those responsible for teaching, since essential information regarding diagnosis (e.g. pathology, internal medicine) and management (e.g. medical oncology, surgery) of tumor entities is typically taught later in the curriculum and most students thus lack a basic understanding necessary to benefit fully from teaching in radiation oncology. The median number of teaching units per student and study program is about 19. The majority of teaching managers are convinced that radiation oncology is not sufficiently represented in the curriculum, and recommend that an average of 5 additional teaching units per student would be useful. Interaction between students and radiation oncology patients takes place at just under half of the sites. Often, either entity-specific teaching or basic radiation therapy is underrepresented. According to a free text entry, two thirds of respondents believe an increase in longitudinal and interdisciplinary radiation oncology teaching is necessary. Elective courses in radiation oncology are highly variable between sites. Most already offer a separate final medical year (practical year, PJ) or quarter, as well as participation in a separate elective, but in many hospitals the subject is to be further expanded. Virtual instruction, mandated by the COVID 19 pandemic and still mostly provisional, occurred across the board. In principle, there is a goal to continue and further develop virtual instruction in various formats in the future.

Overall, there is a need to map radiation oncology teaching in a longitudinal, interdisciplinary, oncology framework. Thus, the basics of radiation therapy should already be included in the first four semesters.

### Recommendations of the academic consortium radiation oncology (*AG Akademische Radioonkologie*) for the scope of teaching

Based on the survey, the Academic Consortium Radiation Oncology of the German Society of Radiation Oncology (*AG Akademische Radioonkologie der* DEGRO) recommends that from the students’ perspective, there should be at least 25 mandatory teaching units for medical students, which should be taught as part of the core curriculum. These include:

- 5 teaching units on basics of radiation therapy (introduction, radiobiology, radiation physics/radiation protection, target volume concept/anatomy, educational discussion) ideally in the form of seminars (groups of up to 20 students in the context of physics, biology, physiology, anatomy and the most important entities).
- 5 teaching units on the basics of clinical radiotherapy (introduction, radiation chemotherapy/immunotherapy, devices, teletherapy/brachytherapy, target volume concept/imaging, radiation planning, shared decision making/side effects/supportive therapy) optimally in the form of seminars (groups of up to 20 students, possibly with a preparatory course/teaching videos followed by classroom sessions).
- 10 teaching units on major tumor entities/indications (gynecologic oncology, uro-oncology, gastrointestinal oncology, thoracic oncology, neuro-oncology, ear, nose and throat (ENT) tumors, hemato-oncology, palliative care, benign indications) preferably in the form of innovative interdisciplinary hybrid events (lectures, seminars, flipped-classroom, case discussion rounds in groups of 20 students or more).
- 3 teaching units with radiation therapy patient contact potentially in the form of bedside teaching followed by case discussion (clinical examination, documentation of disease data and treatment, radiation planning and imaging, side effects and supportive therapy, management and procedures).
- 2 teaching units on radiation therapy/obtaining informed consent (structure/small group exercises).

In addition, radiation therapy and radiation oncology should be included as broadly as possible in the specialization area (20% of the curriculum), and faculties should also offer extensive options in the core curriculum. This is crucial for the promotion, appeal, and advancement of radiation therapy and radiation oncology among future physicians. Optional teaching should include the following offerings:

- Independent quarter of the final medical year (practical year, PJ) (individual students) as well as clinical traineeships.
- Radiation oncology elective (approx. 25 h of instruction) with creation of more in-depth skills (contouring, case discussions, radiation planning, seminar/small group).
- Participation as part of the 1–2-week elective block internship in a clinical hands-on subject.
- Supervision in the context of the newly created mandatory 12-week scientific paper between the 1st and 2nd state examinations (1–3 students per thesis).
- In-depth area: e.g. offering an elective interdisciplinary oncological discussion group: for example, visits to real tumor boards with preparation by the students and professional debriefing (approx. 10 teaching hours/small groups).

## At which point and in which teaching format can these topics be anchored in the new curriculum?

Due to the necessary basic medical knowledge to understand radiation therapy, it still makes sense to offer the majority of radiation therapy and radiation oncology teaching during the 5th–10th semesters; however, individual elements such as radiobiology, radiation physics, or oncological interviewing can also be integrated into the first 4 semesters [13]. In particular, the area of specialization, as selected by students, allows significantly more intensive teaching during electives, internships, science projects, etc., with the inclusion of a larger number of students.

Innovative teaching concepts and the virtual medical teaching have proven to be effective and are also desired according to student feedback [8, 14–16]. Despite the mostly provisional offerings during the COVID pandemic, these formats will gain importance in future curricula. In the future, hybrid courses consisting of virtual and face-to-face courses, self-study, lecture, seminar, patient teaching, and case discussion will be interlinked [17, 18]. In principle, very complex hybrid formats lend themselves to the teaching of radiation oncology due to its highly interdisciplinary nature and the linking of basic and clinical knowledge as well as competency-based skills [19]. Ultimately, the specific design of radiation oncology teaching at the respective departments depends primarily on the individual commitment of radiation oncologists, interdisciplinary collaboration, and the ultimate design of the curriculum. Since the new licensing regulations will presumably be passed by the Federal Council (Bundesrat) by mid-2021 or 2022 at the latest, the implementation of the new curriculum is already underway or will begin promptly at the individual universities. Various task forces are usually formed for this purpose. It is essential that those responsible for teaching work promptly, intensively, and actively to integrate and expand radiation therapy and radiation oncology teaching at their universities. An example of radiation oncology teaching in the medical curriculum based on the new ÄApprO is summarized in Table 2.

**Table 2:** Example of radiation oncology teaching in the medical curriculum based on the new ÄApprO

Semester	1.–4	5.–10	11.–12
*Mandatory teaching (core curriculum)*	*5* *UE* *Fundamentals of radiation*	5 UE clinical radiation therapy	–
		10 UE interdisciplinary radiation oncology	
		3 UE bedside teaching/case discussion	
		2 UE medical interviewing	
*Optional teaching (core curriculum)*	2 UE oncological interviewing	Elective subject radiation oncology	Quarter of final medical year (PJ)
		Interdisciplinary tumor conference	
		Scientific paper	
		Clinical clerkships	
*Specialization in oncology*	Case conferences, bloc internships, scientific work, OSCE		–

*UE* teaching unit (45 min), *PJ* practical year, *OSCE* objective structured clinical examination

## What are the arguments for establishing radiation therapy teaching in the respective faculties?

The survey presented in this concept paper can be used to argue for a Germany-wide standard with reference to the recommended scope of teaching. In principle, there is a claim for radiation therapy teaching via the integration of radiation therapy and radiation oncology into the cross-sectional subject “imaging techniques, radiation treatment, radiation protection” (QS) [7]. Furthermore, general reference can be made to section VII.4.16.1.2 “explain the basic principle of radiation therapy and give indications, contraindications, and relevant clinical examples” and to section VII.4.16.1.4 “explain the principles of radiochemotherapy” of the GK (identical in wording to NKLM) [5, 6]. In addition, numerous interdisciplinary competencies are explicitly listed, such as in the NKLM under 16.1.1.7 “explain, critically discuss, and apply the principles of interdisciplinary as well as interprofessional therapy using concrete examples”. Here, among other subjects, radiation therapy is also explicitly mentioned in the application example and in the performance record.

Fundamentally, there is great potential for establishing radiation therapy in the common tumor entities that are most heavily weighted in the GK. In some cases, these are already taught in the first four semesters (V1) and are therefore potentially also queried by the IMPP in the first state examination [6]. With reference to the longitudinal and interdisciplinary focus of the new curriculum, the integration of radiation oncology into the teaching of these entities should be mandatory. In the case of rare tumors, treated primarily with chemoradiotherapy (e.g. anal carcinoma, vulvar carcinoma), an additional argument can be made for appropriate teaching units based on the outstanding therapeutic importance of chemoradiotherapy.

However, it should be noted that the current versions of the NKLM and the GK are not definitive final documents and are currently still being adapted and further developed, e.g., by the input of representatives of various medical societies.

Overall, the increasing competency-based and multidisciplinary nature of the new curriculum potentially offers more opportunities for meaningful teaching of radiation oncology in the interdisciplinary setting outside of QS imaging.

## Conclusion

The study of medicine will undergo far-reaching reforms due to the new ÄApprO, and the respective curriculum is being designed currently or will be in a timely manner at the respective faculties. Radiation oncology is an integral part of modern interdisciplinary tumor treatment and should be represented accordingly in the curriculum. It is critical that the respective radiation oncology teaching faculty and the heads of the departments for radiation therapy and radiation oncology become actively involved in the curriculum redesign process at their respective faculties immediately.

Figs. 2 and 3 summarize the main three questions regarding the scope, potential integration, and associated rationale for mandatory and optional radiation oncology teaching in the new curriculum. Both the ÄApprO and the current versions of the NKLM and the GK were examined with respect to the occurrence of radiation therapy and its closely related topics and subjects, and the corresponding competency levels and priorities contained therein were presented to support the argument for the integration of radiation therapy and radiation oncology teaching.

**Fig. 2:**
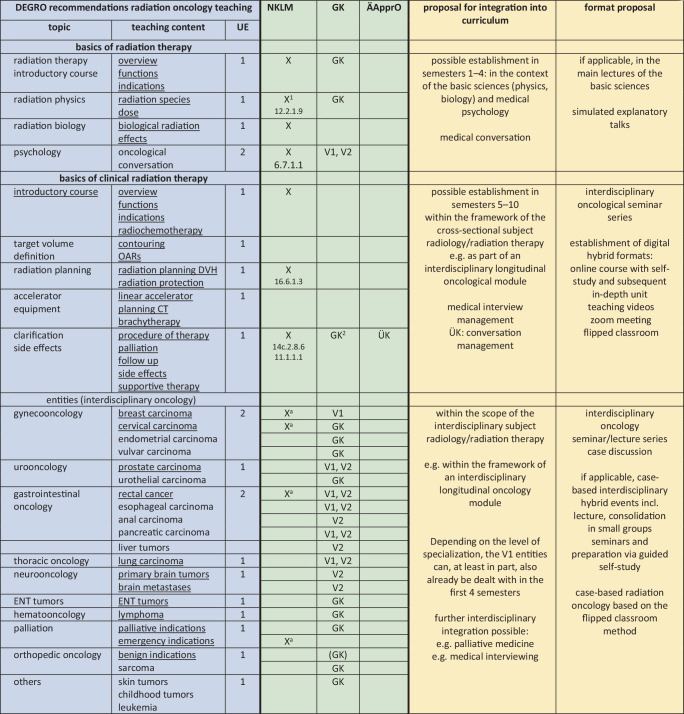
DEGRO recommendations for the establishment of mandatory radiation oncology teaching in the new medical curriculum. *NKLM* National Competence-Based Learning Objectives Catalogue for Medicine 2.0, *GK* mentioned in the subject catalogue of the IMPP, *ÄApprO* Ärztliche Approbationsordnung, *ÜK* higher level competence, *UE* teaching unit (45 min), *underlined* should be taught without fail, *ZV* target volume, *X* radiation therapy explicitly mentioned, *a* mentioned as cross-reference or example, *V* generally prioritized topic, *V1* diseases of the focus disease network semester 1–4, *V2* in-depth study planned in semesters 5–10, *1* radiation protection explicitly mentioned, *2* radiation enteritis explicitly mentioned

**Fig. 2:**
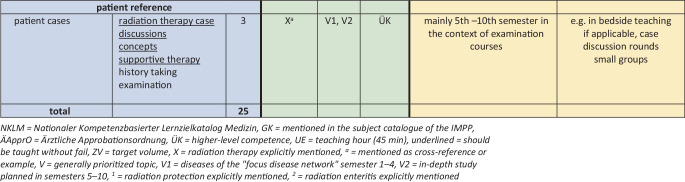
(continued)

**Fig. 3:**
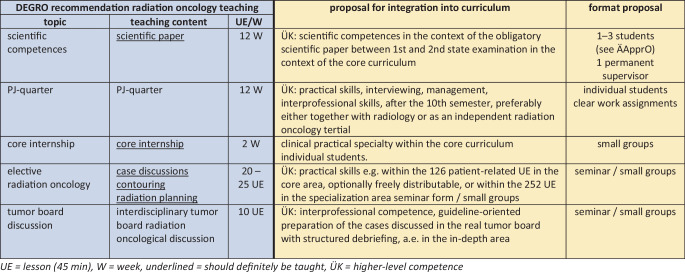
DEGRO recommendations for the establishment of the facultative radiation oncology teaching (core curriculum and specialization area) in the new medical curriculum. (*UE* lesson 45 min, *W* week, *underlined* should definitely be taught, *ÜK* higher level competence)
